# Recurrence and visual prognostic factors of polypoidal choroidal vasculopathy: 5-year results

**DOI:** 10.1038/s41598-021-00904-4

**Published:** 2021-11-03

**Authors:** Joo Young Kim, Woo Young Son, Rae Young Kim, Mirinae Kim, Young Gun Park, Young-Hoon Park

**Affiliations:** 1 Department of Ophthalmology and Visual Science, Seoul St. Mary’s Hospital, College of Medicine, The Catholic University of Korea, 222 Banpo-daero, Seocho-gu, Seoul, 06591 Korea; 2 Catholic Institute for Visual Science, College of Medicine, The Catholic University of Korea, Seoul, Korea

**Keywords:** Eye diseases, Macular degeneration, Retinal diseases, Vision disorders

## Abstract

This retrospective study aimed to evaluate the factors affecting recurrence and visual prognosis in patients with treatment-naïve subfoveal polypoidal choroidal vasculopathy (PCV). Patients who had received three consecutive intravitreal injections of ranibizumab or aflibercept and had reached remission were enrolled. They were divided into a group without recurrence (group 1, 26 eyes) and a group with recurrence (group 2, 121 eyes) and followed up for at least 5 years. Patients in group 2 received additional treatment for worsening. Logistic regression analysis revealed that a young age of onset (*P* = 0.001), high choroidal vascularity index (CVI; *P* = 0.019), and presence of choroidal vascular hyperpermeability (CVH; *P* = 0.037) were associated with a low risk of recurrence. Multiple regression analysis revealed that recurrence (*P* = 0.001), greatest linear dimension (*P* = 0.003), and polyp configuration (single or cluster; *P* = 0.043) were associated with final visual acuity. Patients without recurrence had a lower age of onset and higher CVI than those with recurrence, and they tended to have CVH. In addition, patients with recurrence, large lesion, and cluster polyps had worse final visual acuity than those without these factors. CVI and CVH may be used to predict recurrence of PCV.

## Introduction

Polypoidal choroidal vasculopathy (PCV), named by Yannuzzi in 1990^1^, is characterized by abnormal branching networks of vessels observed on indocyanine green angiography (ICGA)^2,3^. It was thought to be a subtype of age-related macular degeneration (AMD); however, several studies have revealed differences in clinical features and genetics^4,5^. Specifically, PCV has a higher incidence in Asians, a lower bilaterality (bilaterality: 5.9–24.1%), and occurs at a younger age than AMD^4,6^. Although most European patients with PCV are women, most Asian patients are men^7,8^.

Various treatment options for PCV exist, such as photodynamic therapy (PDT), intravitreal injection of anti-vascular endothelial growth factor (VEGF), PDT and anti-VEGF combination therapy, and focal laser. The EVEREST-II study compared ranibizumab monotherapy and combination therapy with PDT and showed that combination therapy required fewer injections and produced better visual outcomes than monotherapy^9^. The PLANET study did not find any potential benefits when rescue PDT was added to aflibercept monotherapy^10^. In the real world, anti-VEGF monotherapy is widely used as the first-line treatment due to inability to perform PDT or due to the disadvantages and costs of PDT.

Recurrence of PCV is known to be common. Akaza et al.^11,12^ reported a 2-year recurrence rate of 64% and 3-year recurrence rate of 77%. Kang et al.^13^ reported a 5-year recurrence rate of 78.6%. However, there is no consensus regarding the visual prognostic or recurrence-related factors of PCV.

In this study, we investigated the baseline characteristics of patients with treatment-naive subfoveal PCV, observed for more than 5 years, and classified them according to recurrence and final visual acuity to determine visual prognostic or recurrence-related factors.

## Results

### Factors related to recurrence

A total of 147 eyes of 147 patients were studied (group 1: 26 patients; group 2: 121 patients). The mean follow-up period was 86.04 ± 24.39 months in group 1 and 79.68 ± 27.32 months in group 2. The mean age at diagnosis of group 1 (62.58 ± 6.90 ​​years) was statistically significantly lower than that of group 2 (67.74 ± 7.15 years; *P* = 0.001). The mean choroidal vascularity index (CVI) of group 1 (65.55 ± 2.96) was higher than that of group 2 (63.21 ± 2.73; *P* = 0.003). Group 1 had more patients with choroidal vascular hyperpermeability (CVH) than group 2 (*P* = 0.046). There were no between-group differences in sex, lens status, spherical equivalent, baseline best-corrected visual acuity (BCVA), largest polyp height, largest polyp diameter, polyp configuration, central foveal thickness (CFT), subfoveal choroidal thickness (SFCT), greatest linear dimension (GLD), first treatment, and presence of subretinal hemorrhage (Table 1).

**Table 1:** Between-group differences in clinical features and characteristics of patients with polypoidal choroidal vasculopathy.

	Group 1 (n = 26)	Group 2 (n = 121)	*P* value^†^
Age at diagnosis	62.58 ± 6.90	67.74 ± 7.15	0.001*
Sex	17:9	89:32	0.399
Lens status Phakic: Pseudophakic	24:2	104:17	0.381
Spherical equivalent	− 0.21 ± 1.27	0.26 ± 1.53	0.080
Baseline BCVA, logMAR	0.35 ± 0.27	0.45 ± 0.25	0.151
Largest polyp height	191.57 ± 80.26	234.99 ± 112.76	0.130
Largest polyp diameter	210.27 ± 123.30	228.27 ± 102.63	0.162
Polyp configuration Single: Cluster	13:13	40:81	0.103
Central foveal thickness	340.30 ± 75.62	373.13 ± 103.13	0.266
Subfoveal choroidal thickness	287.10 ± 121.79	279.87 ± 105.11	0.962
Choroidal vascularity index	65.55 ± 2.96	63.21 ± 2.73	0.003*
Choroidal vascular hyperpermeability Present: Absent	17:9	53:68	0.046*
Greatest linear dimension	1972.11 ± 543.14	2450.68 ± 1139.06	0.059
First treatment Ranibizumab: Aflibercept	18:8	86:35	0.851
Subretinal hermorrhage Present: Absent	4:22	34:87	0.374

Values are presented as mean ± standard deviation unless otherwise indicated.

BCVA, best-corrected visual acuity; logMAR, logarithm of the minimum angle of resolution.

*P < 0.05.

^†^*P* values were calculated using the Chi-square test for categorical variables and the Mann–Whitney test for continuous variables.

On univariate analysis, a young age (*P* = 0.014), small largest polyp diameter (*P* = 0.048), small CFT (*P* = 0.039), high CVI (*P* = 0.031), and presence of CVH (*P* = 0.044) were found to be associated with a low risk of recurrence. On multivariate analysis, a young age (*P* = 0.001), high CVI (*P* = 0.019), and presence of CVH (*P* = 0.037) were associated with a low risk of recurrence (Table 2).

**Table 2:** Logistic regression analysis for recurrence of polypoidal choroidal vasculopathy.

Independent variables	Univariate analysis			Multivariate analysis		
	OR	95% CI	*P* value	OR	95% CI	*P* value
Age at diagnosis	1.099	1.020–1.185	0.014*	1.116	1.047–1.190	0.001*
Sex	1.257	0.452–3.495	0.661			
Lens status Phakic: Pseudophakic	10.484	0.190–577.173	0.251			
Spherical equivalent	1.535	0.740–3.182	0.250			
Baseline BCVA, logMAR	0.251	0.007–8.522	0.443			
Largest polyp height	0.999	0.987–1.012	0.901			
Largest polyp diameter	1.012	1.000–1.024	0.048*	1.009	1.000–1.018	0.064
Polyp configuration Single: Cluster	2.899	0.454–18.488	0.260			
Central foveal thickness	1.019	1.001–1.037	0.039*	1.010	1.000–1.021	0.055
Subfoveal choroidal thickness	0.996	0.985–1.008	0.529			
Choroidal vascularity index	0.640	0.427–0.959	0.031*	0.687	0.503–0.940	0.019*
Choroidal vascular hyperpermeability Present: Absent	0.045	0.002–0.920	0.044*	0.158	0.028–0.892	0.037*
Greatest linear dimension	1.001	1.000–1.002	0.175	1.001	1.000–1.002	0.092
First treatment Ranibizumab: Aflibercept	0.400	0.071–2.237	0.297			
Subretinal hermorrhage	0.392	0.024–6.313	0.509			

BCVA, best-corrected visual acuity; logMAR, logarithm of the minimum angle of resolution.

*P < 0.05.

Of the patients in group 2, 55 received only anti-VEGF injections, 48 received PDT once in addition to anti-VEGF injections, 14 received PDT twice, and 4 received PDT three times.

### Factors related to visual prognosis

The average logarithm of the minimal angle of resolution (logMAR) BCVA at baseline and at 3 months after the first treatment (three consecutive injections of an anti-VEGF agent) did not differ between the two groups (*P* = 0.104 and *P* = 0.285, respectively). In contrast, the average logMAR BCVA of group 1 was better than that of group 2 from 1 year to the last follow-up visit (1 year, *P* = 0.031; 2 years, *P* = 0.010; 3 years, *P* = 0.002; 4 years, *P* < 0001; 5 years, *P* = 0.001; last visit, *P* < 0.001). Repeated-measures analysis of variance (ANOVA) revealed a difference in the BCVA over time in both groups (*P* < 0.001; Table 3).

**Table 3:** Changes in best-corrected visual acuity over time between group 1 and group 2.

BCVA, logMAR	Group 1 (n = 26)	Group 2 (n = 121)	*P* value^†^
Baseline	0.35 ± 0.27	0.45 ± 0.25	0.104
3 months	0.30 ± 0.28	0.41 ± 0.30	0.285
1 year	0.23 ± 0.21	0.36 ± 0.31	0.031*
2 years	0.23 ± 0.23	0.41 ± 0.37	0.010*
3 years	0.23 ± 0.23	0.44 ± 0.36	0.002*
4 years	0.22 ± 0.22	0.50 ± 0.41	< 0.001*
5 years	0.20 ± 0.23	0.59 ± 0.50	< 0.001*
Last visit	0.19 ± 0.22	0.76 ± 0.65	< 0.001*
Repeated measures ANOVA			< 0.001*

Values are presented as mean ± standard deviation unless otherwise indicated.

BCVA, best-corrected visual acuity; logMAR, logarithm of the minimum angle of resolution.

*P < 0.05.

^†^*P* values were calculated using the Mann–Whitney test for one time point and repeated measures ANOVA over time.

According to Pearson's correlation analysis, the final visual acuity was lower in patients who were older at the time of diagnosis (*P* = 0.006). In addition, patients with recurrence (*P* = 0.001), cluster polyps (*P* = 0.013), and greater GLD (*P* = 0.001) had worse final visual acuity than those without these factors. According to multiple regression analysis, patients with recurrence (*P* = 0.001), cluster polyps (*P* = 0.043), and greater GLD (*P* = 0.003) were found to have worse final visual acuity (Table 4).

**Table 4:** Prognostic factors affecting the final visual acuity in polypoidal choroidal vasculopathy.

Independent variables	Pearson’s correlation analysis		Multiple regression analysis		
	Coefficient	*P* value	Standardized beta coefficient	t	*P* value
Age at diagnosis	0.249	0.006*	0.160	1.826	0.070
Sex	0.041	0.657	0.013	0.147	0.884
Lens status Phakic: Pseudophakic	0.038	0.682	0.004	0.041	0.967
Spherical equivalent	0.039	0.669	0.014	0.161	0.872
Baseline BCVA, logMAR	0.173	0.057	0.138	1.589	0.115
Recurrence	0.332	0.001*	0.286	3.350	0.001*
Largest polyp height	0.002	0.980	− 0.040	− 0.452	0.652
Largest polyp diameter	0.024	0.794	− 0.043	− 0.485	0.628
Polyp configuration Single: Cluster	0.226	0.013*	0.177	2.041	0.043*
Central foveal thickness	0.081	0.377	0.024	0.268	0.789
Subfoveal choroidal thickness	− 0.108	0.242	− 0.094	− 1.085	0.280
Choroidal vascularity index	− 0.062	0.503	0.038	0.419	0.676
Choroidal vascular hyperpermeability Present: Absent	0.024	0.794	0.091	1.028	0.306
Greatest linear dimension	0.307	0.001*	0.258	3.021	0.003*
First treatment Ranibizumab: Aflibercept	− 0.087	0.343	− 0.069	− 0.788	0.433
Subretinal hermorrhage	0.127	0.167	0.082	0.939	0.350

BCVA, best-corrected visual acuity; logMAR, logarithm of the minimum angle of resolution.

*P < 0.05.

### Correlations of choroid-related parameters

According to Pearson's correlation analysis, CVI was significantly correlated with CVH (*P* = 0.009). However, SFCT was not significantly correlated with CVI and CVH (*P* = 0.841 and *P* = 0.127, respectively; Table 5).

**Table5:** Correlations of choroid-related parameters.

Coefficient/*P* value	Subfoveal choroidal thickness	Choroidal vascular index	Choroidal vascular hyperpermeability
Subfoveal choroidal thickness		0.018/0.841	− 0.140/0.127
Choroidal vascular index	0.018/0.841		0.237/0.009*
Choroidal vascular hyperpermeability	− 0.140/0.127	0.237/0.009*	

*P* values were calculated using Pearson’s correlation analysis.

*P < 0.05.

## Discussion

We investigated the factors associated with recurrence and final visual prognosis in patients with PCV who were observed for more than 5 years. Patients with a young age of onset and high CVI and those who had CVH had a low risk of recurrence. This is the first study to show that CVI and CVH are associated with recurrence in patients with PCV.

The primary pathophysiology of PCV is choroidal congestion and leakage, which is presumed to lead to overall choroidal thickening^14^. This choroidal thickening is due to choroidal vessel dilatation, which increases the luminal area^15,16^. The outer choroidal layer thickness increases, and the Haller layer is thickened along the large vessel^14^.

Patients with PCV have a greater SFCT than patients with AMD or healthy individuals; meanwhile, they have a lower CVI than healthy individuals^17^, with no statistically significant difference from that in patients with AMD^18^. In general, SFCT is more variable than CVI and is affected by physiological and ocular factors, which do not affect CVI, making it a more stable marker for choroidal disease^19^.

Gupta et al.^15^ used a technique other than CVI to measure vascular area and classified PCV into two subtypes according to choroidal vascular characteristics. Typical PCV has large SFCT and choroidal vascular area, and choroidal vessel dilatation is an important pathophysiology of this subtype. On the other hand, PCV with relatively small SFCT has smaller vascular area than typical PCV, and has characteristics similar to neovascular AMD; therefore, it was termed polypoidal choroidal neovascularization (CNV). In this subtype, choroidal vessel dilatation is not a major pathophysiology, and the prognosis has been predicted to be similar to that of neovascular AMD.

Cheung et al.^20^ compared neovascular AMD and PCV and reported that although patients with neovascular AMD receive a significantly greater number of anti-VEGF injections per year than those with PCV (4.51 times vs. 3.43 times), there was no difference in final visual acuity. Ting et al.^21^ reported that the average age of onset of PCV was 67.6 years, which was younger than that of neovascular AMD (72.5 years), and patients with PCV received fewer anti-VEGF injections per year after onset than those with neovascular AMD (3.9 times vs. 5.6 times). In both these studies, patients with PCV required fewer injections than those with neovascular AMD. Considering that patients in group 1 have characteristics similar to those of typical PCV (large SFCT and low age of onset) and those in group 2 have characteristics similar to those of polypoidal CNV (frequent recurrence), our findings are consistent with those of previous studies and indicate that patients with PCV require fewer injections than those with neovascular AMD.

Liu et al.^22^ classified PCV into two groups according to the presence or absence of CVH. When CVH was present, CVI tended to be high. CVH occurs due to dilatation of the choriocapillaris and choroidal vein^16^, and an increase in the luminal area compared to the total choroidal area results in an increase in CVI; this may explain the association between CVH and CVI. In this study, the CVI was higher in group 1 than in group 2, and there were more patients with CVH in group 1 than in group 2. In addition, as shown in Table 5, CVH occurred more often when the CVI was high, which was consistent with the findings of Liu et al.. Group 1, which has characteristics similar to typical PCV, had a higher CVI and no recurrence. On the other hand, group 2, which have characteristics similar to neovascular AMD, had lower CVI, more frequent recurrence, and required more injections.

In this study, patients with recurrence, cluster polyps rather than single polyps, and larger GLD had worse final visual acuity than those without these factors. Uyama et al.^23^ explained that cluster polyps were associated with a poor visual prognosis because of the high risk of leakage and bleeding. Lee et al.^24^ indicated that rather than treatment methods, initial visual acuity, lesion size, patient age, and polyp location were related to visual prognosis. Tsujikawa et al.^25^ reported that patients with small vascular lesions and GLDs had a good visual prognosis because they experienced few complications that could cause severe visual loss and disease progression.

In this study, recurrence and final visual acuity did not differ according to the drug administered in the first treatment. This was consistent with the findings of Cho et al.^26^, who found no difference in final visual acuity after 1 year between PCV patients treated with ranibizumab and aflibercept.

Yanagi et al.^27^ reported that patients with CVH had better visual outcomes than those without CVH. Additionally, among patients who underwent combination therapy, those with CVH received fewer injections than those without CVH. In this study, a higher proportion of patients in group 1 had CVH, and they had a higher CVI than those in group 2; additionally, they had no recurrence and required fewer injections than those in group 2. Patients without recurrence had better final visual acuity than those with recurrence, but CVI and CVH were not correlated with final visual acuity. There was no between-group difference in visual acuity at baseline and at 3 months after the first treatment, but group 1 had better visual acuity than group 2 from 1 year after the baseline until the last follow-up visit. Although we did not investigate the number of recurrences in group 2, this group includes patients with persistent fluid that are no longer effective against anti-VEGF, disciform scar, vision-threatening complications such as subretinal hemorrhage and retinal pigment epithelial tears. Severe visual impairment may occur during the clinical course after recurrence and repeated treatment. Although CVI and CVH are associated with recurrence after the first 3 months of treatment, they may not be predictive of visual prognosis due to changes in the clinical course over a 5-year period.

One limitation of the study is that the number of patients in group 1 was relatively small, probably because more patients in this group discontinued follow-up than in group 2 as there was no recurrence and their condition was stable. In addition, the evaluation of CVH using ICGA is subjective and depends on the evaluator. Furthermore, group 2 was treated with anti-VEGF injections or PDT after recurrence, but the type and number of treatments administered after recurrence were not reflected in the analysis of the final visual acuity.

In conclusion, patients without recurrence had lower age of onset and higher CVI than those with recurrence, and they tended to have CVH. In addition, patients with recurrence, cluster polyps, and large lesions had worse visual acuity than those without these factors. Currently, the calculation of CVI requires a complex process; however, if this process is automated, CVI and CVH can be used to predict recurrence in patients with PCV. Patients with poor visual prognostic factors may need careful observation. A large-scale prospective study is needed in the future to confirm the results of this study.

## Methods

### Study population

This retrospective cohort study was conducted using data from medical records. This study adhered to the tenets of the Declaration of Helsinki. Institutional Review Board/Ethics Committee approval was obtained from Seoul St. Mary’s Hospital, The Catholic University of Korea. The institutional review board of Seoul St. Mary’s Hospital, The Catholic University of Korea waived the need for informed consent due to the retrospective nature of the study (KC21RASI0284). Treatment-naive Korean patients diagnosed with subfoveal PCV at Seoul St. Mary's Hospital between April 1, 2010 and March 31, 2016 who followed up for more than 5 years after diagnosis and achieved complete remission after the first treatment were included. The first treatment was limited to three consecutive intravitreal injections of ranibizumab (0.5 mg; Lucentis, Genentech-Roche, South San Francisco, California, USA) or aflibercept (2 mg; Eylea, Bayer HealthCare, Berlin, Germany) every month. PCV was diagnosed as polypoidal dilatation with or without a branching vascular network on ICGA^2^. Patients who received PDT as the first treatment or a combination of PDT and anti-VEGF agents were excluded. Patients with concurrent neovascular AMD or ocular disease affecting visual acuity, those who had been treated at other hospitals or had previously undergone vitrectomy, and those with incomplete records were excluded.

### Treatment

After three injections of ranibizumab or aflibercept, patients received additional treatment whenever intraretinal fluid or subretinal fluid involving the fovea was observed. Additional treatment was primarily an intravitreal injection of the anti-VEGF agent, that had been injected as the first treatment; if it was not effective, the injected drug was changed or PDT was administered. Bevacizumab (1.25 mg; Avastin, Genentech-Roche) was also administered to patients who were unable to receive ranibizumab or aflibercept according to Korean insurance guidelines. Fluorescein angiography (FA) and ICGA were repeated when new lesions were thought to have occurred.

**Figure 1:**
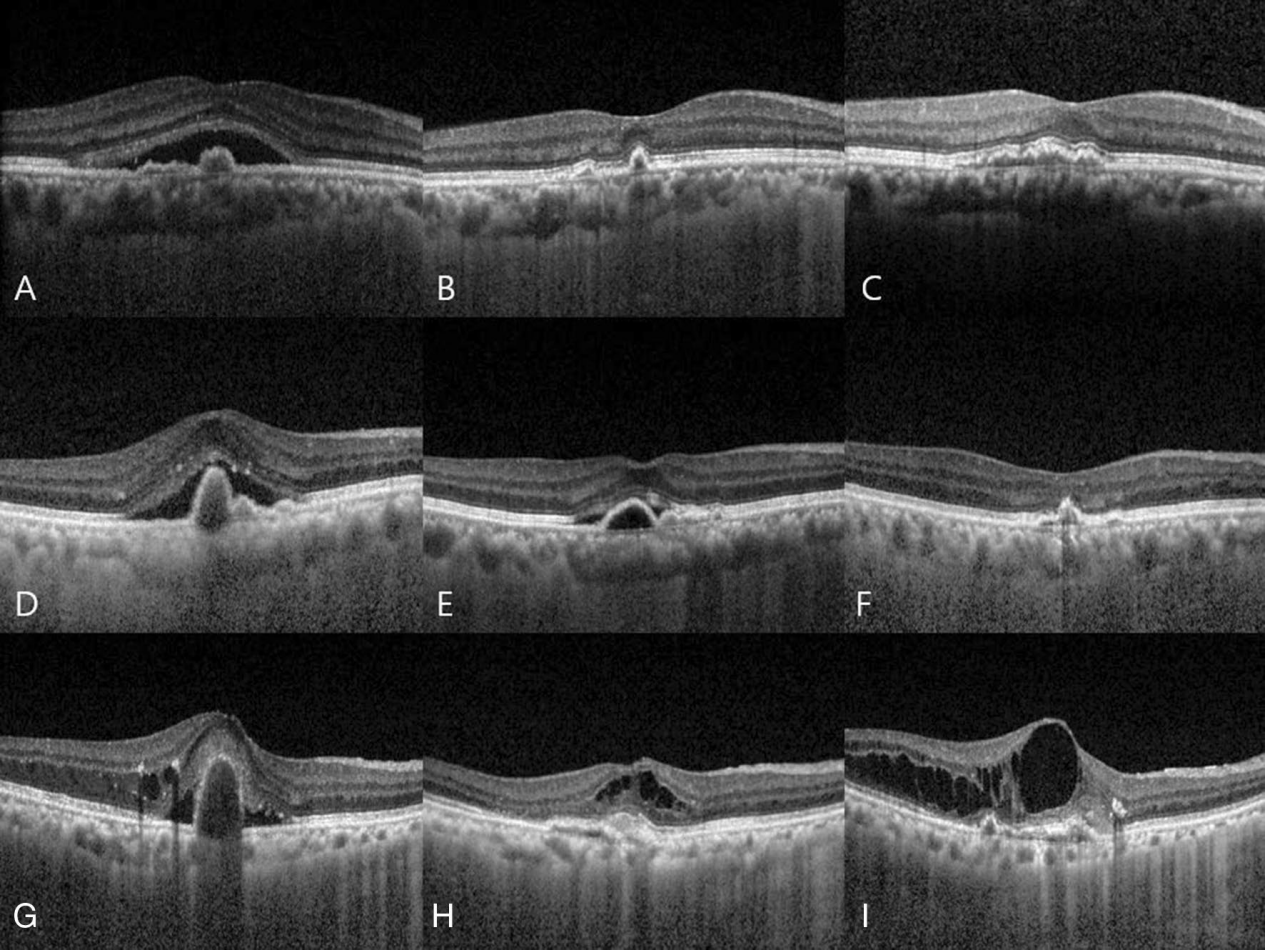
Representative optical coherence tomography images of each groups. (**A**–**C**) Images of a 68-year-old man from group 1 with a baseline BCVA of 10/20 (**A**). Remission is reached after the administration of three doses of ranibizumab, and the BCVA is 12/20 at 6 months from onset (**B**). The BCVA at 5 years from onset is 16/20 (**C**). (**D**–**F**) Images of a 69-year-old man from group 2 with a baseline BCVA of 12/20 (**D**). Remission is reached after the administration of three doses of aflibercept, but recurrence occurs. The BCVA is 10/20 at 6 months from onset (**E**). Repeated recurrence and remission occur, and the patient receives 13 additional aflibercept injections. The BCVA is 12/20 at 5 years from onset (**F**). (**G**–**I**) Images of a 70-year-old woman from group 2 with a baseline BCVA of 10/20 (**G**). Remission is reached after the administrations of three doses of ranibizumab, but recurrence occurs. The BCVA is 10/20 at 6 months from onset (**H**). Repeated recurrence and remission occur, and the patient receives 44 additional anti-vascular endothelial growth factor injections (ranibizumab, aflibercept, and bevacizumab) and two sessions of photodynamic therapy. The BCVA is 2/20 7 years after onset (**I**). BCVA: best-corrected visual acuity.

Patients who underwent the abovementioned treatment were divided into two groups for analysis. Patients who achieved remission after the first treatment and did not require additional treatment for more than 5 years were assigned to group 1. Those who did not receive further treatment due to macular scarring after the first treatment were excluded from group 1. Patients who achieved remission after the first treatment, but had one or more recurrences, who received additional treatment were assigned to group 2. Representative cases of each group are presented in Fig. 1.

### Outcome measures

All patients underwent BCVA, refraction, intraocular pressure measurements, color fundus photography, slit-lamp microscopy, dilated fundus examination, spectral-domain optical coherence tomography (OCT; Heidelberg Engineering, Heidelberg, Germany) with enhanced depth imaging (EDI), FA, and ICGA (Heidelberg Retina Angiograph-2, Heidelberg Engineering) at baseline. In addition, they underwent BCVA and intraocular pressure measurements, slit-lamp microscopy, dilated fundus examination, and spectral-domain OCT with EDI at every follow-up visit to evaluate lesion activity. Patients who received additional treatment due to recurrence were followed up every 4 weeks, and when treatment was not needed, the follow-up period was extended to 8 weeks or more.

CFT, SFCT, and largest polyp height were measured on baseline OCT. The presence or absence of subretinal hemorrhage was also determined using OCT. CFT, which is the thickness of the 1-mm central area, was automatically measured using OCT. SFCT was defined as the distance between the Bruch membrane and the choroid-scleral border at the fovea and was measured using horizontal and vertical line scans intersecting the center of the fovea. The largest polyp height was measured using OCT software. CVI was measured on the full-length EDI-mode OCT image using a technique described in a previous study^28,29^.

ICGA using Heidelberg Retina Angiograph-2 software was used to assess polyp location, largest polyp diameter, polyp configuration (single or cluster), GLD, and the presence or absence of CVH at baseline. The largest polyp diameter and GLD were measured using software. CVH was evaluated using a late-phase ICGA image. In cases where it was difficult to evaluate the lesion on ICGA due to massive subretinal hemorrhage, ICGA was performed after subretinal hemorrhage decreased. OCT and ICGA images were reviewed by two retina specialists (J.Y.K. and R.Y.K.), and differences of opinion were resolved through open discussion. In case of disagreement between the two, an expert (Y.H.P.) made the final decision.

### Analysis

BCVA values were converted into logMAR units. Patients who could only count fingers or detect hand motion were assigned logMAR values of 2.0 and 3.0, respectively^30^.

All data were statistically analyzed using the Statistical Package for the Social Sciences software, version 24.0 (SPSS Inc, Chicago, IL, USA). Continuous variables were compared using the Mann–Whitney test, and categorical variables were analyzed using the Chi-square test. Logistic regression analysis was performed to determine the factors influencing recurrence. Repeated-measures ANOVA was used to compare visual acuity over time between the two groups. Pearson’s correlation analysis and multiple regression analysis were performed to determine prognostic factors affecting the final visual acuity. A p-value of < 0.05 was considered statistically significant.

## Data Availability

The datasets analyzed during the current study are available from the corresponding authors on reasonable request.
